# Specialized recall procedures

**DOI:** 10.3758/s13423-025-02744-z

**Published:** 2026-02-17

**Authors:** Lynn J. Lohnas, Sean M. Polyn, Michael J. Kahana

**Affiliations:** 1 Department of Psychology, Syracuse University, Syracuse, New York USA; 2 Department of Psychology, Vanderbilt University, Nashville, Tennessee USA; 3 Department of Psychology, University of Pennsylvania, Philadelphia, Pennsylvania USA

## Abstract

Studies of memory search using the free recall paradigm have advanced our understanding of human memory for more than a century. Here, we review seven specialized recall procedures that depart from methodological orthodoxy: the inter-list repetition procedure, the list-before-last procedure, the recall-by-category procedure, the final-free recall procedure, the overt-rehearsal procedure, the externalized recall procedure, and the spatial-temporal recall procedure. Key empirical findings from each of these procedures have challenged existing theories and led to theoretical advances.

Recall and recognition constitute the two classic procedures for studying explicit, episodic memory. In both procedures, the experimenter asks participants to remember items experienced on a recent list. Although the participant may or may not know that they will be tested on the items, recognition and recall procedures explicitly require participants to consult their memory for an item’s prior occurrence. Students of memory possess considerable familiarity with the classic variants of recognition (e.g., item vs. associative) and recall (e.g., cued recall, serial recall, and free recall). Our focus here is on the free recall task, and in particular, seven specialized procedures that researchers have developed to better understand context-based episodic memory. Before introducing these seven procedures, we briefly describe the standard free recall procedure.

In free recall, participants typically study a list of words, and then, either immediately or following a filled retention interval, they attempt to recall as many words as they can remember in any order. Because participants can recall items without regard to their order of study, the statistical regularities in their recall order offer clues to the organization of memory. These regularities include the serial position of the item produced during recall initiation (i.e., the first recall response): this is typically one of the final list items when recall immediately follows study, and is typically one of the first list items when recall follows a filled delay period. Recall transitions reveal further regularities, including the tendency to cluster recalls based on their temporal similarity (the contiguity effect), semantic similarity, spatial proximity, emotional valence, or other contextual or source attributes. Previous reviews summarize these and other free recall phenomena (Kahana, 2017, 2020; Healey et al., 2019).

Standard variants of free recall include manipulations of encoding task (Hyde & Jenkins, 1969; Long & Kahana, 2017; Polyn et al., 2009; Postman & Adams, 1956), modality of presentation (Murdock & Walker, 1969; Pazdera & Kahana, 2023), presentation rate and list length (Murdock, 1962; Ward et al., 2010), intra-list repetition (Madigan, 1969; Melton, 1970; Siegel & Kahana, 2014), intra-list semantic similarity (Romney et al., 1993), and categorical organization of lists (Pollio et al., 1969; Tulving & Pearlstone, 1966; Ward & Tan, 2023), among many others (Kahana, 2012). Another standard variant involves giving participants multiple trials to learn a list (multi-trial free recall) with list order either being constant or variable across presentations (Klein et al., 2005; Tulving, 1962). Yet another variant, continual-distractor free recall (CDFR), has participants perform distractor tasks between the study of different list items and also at the end of the study list. This procedure has revealed that recency and contiguity effects appear at both short- and long-time scales, arguing against classic dual-store models of free recall (Bjork & Whitten, 1974; Howard & Kahana, 1999; Neath, 1993).

The great diversity of these procedural variations obscures two critical features of free recall. The first concerns the nature of the target list, which is generally the last set of experienced items. Shared semantic attributes rarely define these items; rather, a span of time and a designation of list membership define them. A second canonical feature of free recall concerns the independence of the target list from other prior experiences. In a typical experiment, participants study and attempt to recall multiple lists, with each list being independent of the others (Adrogue et al., 2024). Both of these features differ markedly from real-world uses of memory. First, we rarely face the challenge of recalling items defined solely based on their joint occurrence on a study list. Second, we often seek to recall memories that share attributes other than their belonging to a recent set of experienced items.

**Fig. 1:**
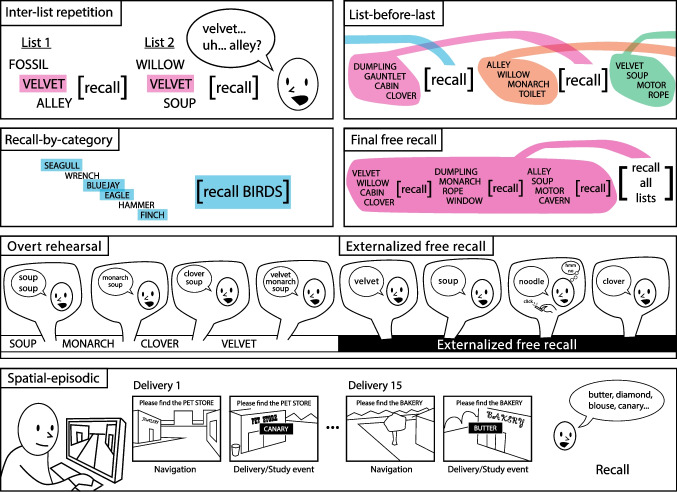
Schematic overview of six specialized recall tasks. Inter-list repetition: Critical items are repeated across lists, challenging a participant’s ability to focus recall on the most recent list. List-before-last: Rather than recall the most recent list of study items, the participant is asked to recall the list studied preceding the most recent list. Recall-by-category: The participant is given a cue prompting them to recall a subset of the studied items. Final free recall: At the end of a session, the participant is asked to recall study items from all of the study-test lists. Overt rehearsal: The participant is encouraged to verbalize any items that come to mind as the study list progresses. Externalized free recall: The participant is encouraged to verbalize any items that come to mind during the free recall period, with a button to indicate when a particular response was not from the study list. Spatial-episodic free recall: The participant navigates to a series of locations in a virtual town, and at each location a study item delivery is revealed. Each series of deliveries is followed by a free recall period

Below, we review seven specialized recall procedures that each deviates in some critical way from the standard procedures described above. In brief, we consider procedures that either alter the recall instructions regarding what the participant must report, or alter retrieval demands in some dramatic way.

Figure 1 depicts each of these procedures:

1. The inter-list repetition procedure alters retrieval demands dramatically, as study-test lists harbor highly similar or identical items. This challenges participants’ ability to focus their retrieval on the just-presented list.
2. The list-before-last procedure alters recall instructions. Following the study of list *i* in a series of lists, participants attempt to recall list $i-1$, while suppressing recall of items from list *i*. This procedure breaks a key feature of standard free recall by having participants target a set of items other than the most recent list. This change arguably makes the task more similar to everyday recall situations outside the laboratory.
3. In the recall-by-category procedure, participants study lists composed of several exemplars from each of several taxonomic categories (e.g., birds or tools). In this procedure, recall instructions are altered, as the experimenter asks for free recall of all exemplars of a cued category. This procedure is a twist on standard categorized free recall in which participants may recall all of the items, both within and across categories, during the recall period (e.g., Pollio et al., 1969).
4. In the final-free recall procedure, participants use standard free recall procedures to study and recall a series of lists. The methodological innovation follows the last study-test lists, when the experimenter asks the participants to recall all items from all studied lists in any order.
5. The overt-rehearsal procedure alters recall instructions dramatically, as participants are asked to verbalize items that come to mind between item presentations, as they study a list for a subsequent recall test. This procedure provides researchers with valuable information on participants’ rehearsal patterns, which in turn predict subsequent recall behavior.
6. The externalized free recall procedure also asks participants to verbalize words that come to mind, but during recall rather than during study. By indicating, with a keypress, which verbalizations they made in error, participants provide valuable information on how they filter or inhibit responses during recall.
7. The hybrid spatial-temporal recall procedure dramatically alters a number of standard features of free recall, in that lists of items unfold in time and space, usually through a cover-task in which participants experience items as they navigate through a virtual environment (see Fig. 7A). This variant shares features of continual-distractor free recall in that participants perform a task (navigation) between each encoded item. Whereas standard free recall places items from a high-dimensional semantic space along a one-dimensional temporal dimension, these tasks place these items within a multivariate spatiotemporal context. In this sense, space acts as a second dimension of context, which may be correlated to various degrees with time but allows for revisiting prior contexts during a single list presentation.

Across many decades, memory researchers have developed a vast array of methodological variants of the free-recall procedure. As such, some procedures fall outside the scope of this paper’s organizational scheme. Throughout the paper, we highlight other notable variant procedures to aid the reader’s further exploration of this rich literature.

## The inter-list repetition procedure

Murdock’s classic (1962) study of the serial position effect in free recall focused the field’s attention on *intra-list* recall phenomena, such as the primacy and recency effects, and on theoretical models aimed at explaining the rich set of phenomena obtained in single-trial free recall. This contrasted with earlier research in the verbal learning tradition, reviewed by Crowder (1976), that emphasized the paired-associate method and used manipulations of similarity and repetition to investigate the effects of proactive interference (PI) and retroactive interference (RI). Although early researchers attracted to the free recall paradigm tended not to focus on classic interference manipulations, it was not long before researchers applied interference ideas and inter-list manipulations to the study of free recall (Postman, 1971).

In standard free recall, there is always a single target list whose items the participant attempts to recall on a given trial. Participants study and recall many such lists throughout an experimental session. Words normally do not repeat across neighboring target lists, which minimizes inter-list interference effects. Nonetheless, performance generally decreases across trials (Kahana et al., 2018), consistent with proactive interference caused by items on prior lists interfering with memory for the target list (Underwood, 1957).

In the inter-list repetition procedure, the target lists include a mixture of items presented on prior lists (repeat items) or items semantically similar to those on prior lists, along with new items (semantically unrelated to prior list items). This repetition of semantic content can produce marked interference. Compared with standard free recall, the inter-list repetition procedure more closely mimics many real-life situations. When shopping for groceries, for example, we must remember our current needs, but in doing so, we must not be overwhelmed by our memories of other recent shopping events that include both overlapping and non-overlapping items. A professor who is teaching two sections of related (or identical) courses must somehow recall what was said in the prior lecture of the target class without being overwhelmed by memories of the other section or the related class. In each of these scenarios, the memory search set often includes items experienced in similar recent contexts, thus taxing our ability to selectively target information experienced within or appropriate to a given context. The inter-list repetition procedure thus provides novel insights into how participants can associate items with cues that support the recall of other items from the proper (but not improper) context.

In an early study of inter-list repetition effects in free recall, Anderson and Bower (1972) asked participants to study lists with many overlapping items across lists. In one experiment, a standard immediate free recall period followed each list. In another experiment, participants attempted to recall any item that had been presented up to that point in the experiment, and for each recalled item, they rated their confidence in that item belonging to the most recent list. They found that as participants encountered more and more repetitions of items across lists, these judgments became less confident. To explain this result, they proposed that participants encode a time-varying signal to differentiate a given word’s occurrence on the target list from the same word’s occurrence on other lists. Participants use this temporal information to restrict their recall to only those items studied in a given context (i.e., the context of the most recent list). According to Bower’s list-discrimination account of inter-list repetition effects (Anderson & Bower, 1972; Sternberg & Bower, 1974), following the retrieval of an item and prior to its production, participants match contextual information retrieved by the item against the context specific to the current list. This account predicts that inter-list repetition will impair the recall of repeated items because the retrieved contexts from earlier presentations will not match the context of the target list.

Anderson and Bower’s manipulation of inter-list repetition in free recall mirrored related literature investigating PI in immediate serial recall of three-item lists. In these procedures, participants studied successive lists featuring items drawn from a single semantic category. After several similar lists, the category switched (Loess, 1967), resulting in a profound ”release” from the effects of PI (Craik & Birtwistle, 1971; Wickens, 1970).

**Fig. 2:**
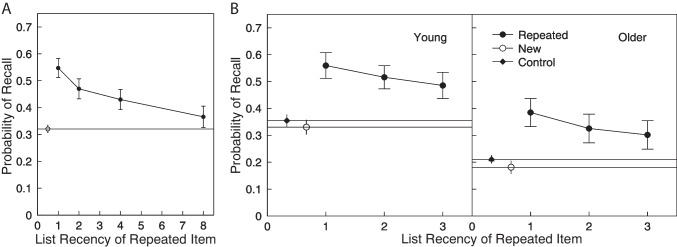
Effects of inter-list repetition on free recall. **A.** When recalling a list of words that includes some words that occurred on prior lists, Zaromb et al. (2006, Experiment 1) found that participants exhibited better recall for repeated items, especially when they occurred on recent prior lists. The probability of recalling once-presented items is designated by a* horizontal line* and an *open circle*. **B.** Younger and older adults exhibited similar inter-list repetition effects (Zaromb et al., 2006, Experiment 2). Here, each list contained items repeated from 1, 2, and 3 lists back. The probabilities of recalling new items in experimental trials with new and repeated items and control trials with no repeated items are designated by the *horizontal lines* with *open* and *filled circles*, respectively. *Error bars* are 95% confidence intervals calculated according to the method of Loftus & Masson (1994). Adapted from Zaromb et al. (2006)

To understand the interaction between memories formed in distinct lists, Zaromb et al. (2006) studied delayed free recall of word lists that included mixtures of new items and repeats from specific earlier lists. They hypothesized that recalling a repeated item would evoke the associated contextual cues from both the target list and the earlier list containing the repeated item. Whereas retrieval of the target-list context would lead to the standard contiguity effect (i.e., the tendency to successively recall items studied in neighboring positions) retrieval of the earlier list context would induce participants to commit prior-list intrusions (PLIs). Consistent with this hypothesis, they found that PLIs occurred more frequently following recall of repeated than novel items (Fig. 2).

Although inter-list repetition led to an increase in PLIs following recall of repeated items, neurologically healthy participants rarely commit PLIs in standard free recall or even when recalling lists with some overlapping items. That participants rarely commit intrusions in free recall underscores the role of source monitoring in episodic retrieval (for a review see Mitchell & Johnson, 2000). In free recall, participants may remember non-presented words to the degree that they possess semantic, phonological, or perceptual similarity to current list items (Roediger & McDermott, 1995). Whether such words appear as intrusions depends on participants’ ability to determine their correct context (sometimes also referred to as the source of the learned information, in this case, whether it occurred in a particular list). The inter-list repetition procedure can thus help researchers investigate the role of source monitoring in recall tasks by activating items learned in the wrong context and challenging participants to filter out those responses.

We have used the term *context* to loosely refer to the set of attributes that distinguish two events experienced at different times and/or places or in association with different background attributes. Theories of free recall have relied heavily on the idea that each item is learned in relation to a set of background contextual attributes. Further, our memory system associates items with the current state of context, thereby allowing the present context to serve as a potent retrieval cue for items experienced in similar contexts. The Search of Associative Memory (SAM) model of free recall (e.g., Raaijmakers & Shiffrin, 1981) served as the leading model of free recall for more than two decades. This model became famous for its mathematical formulation of the interacting roles of short-term and long-term memory, and context played an essential role in the model, providing a mechanism for targeting items based on their list membership. Mensink and Raaijmakers (1988) extended this model to include the idea of a drifting representation of temporal context. Howard and Kahana (2002) introduced a new recursive representation of context and proposed that contextual retrieval guides the evolution of context and helps to explain both recency and contiguity effects in free recall. Kahana, Sederberg, Polyn, and Lohnas extended this theory, implemented as the *Context Maintenance and Retrieval* (CMR) model, to explain a broad array of empirical facts concerning standard variants of the free recall task (Lohnas et al., 2015; Polyn et al., 2009; Sederberg et al., 2008). Lohnas, Polyn, and Kahana extended CMR to account for inter-list effects. However, their model, termed the continuous memory version of the context maintenance and retrieval model or CMR2 model, has not yet been applied to data on the inter-list repetition procedure. Although the model should exhibit temporally graded interference from prior lists, and increased rates of intrusions following recall of repeated items, it is not yet known whether the model will quantitatively account for the full pattern of empirical data generated by this procedure.

## The list-before-last procedure

Whereas the inter-list repetition procedure manipulates proactive interference (PI), the list-before-last (LBL) procedure manipulates retroactive interference (RI). Rather than asking participants to recall the just-presented list in the face of interference from prior lists, the LBL procedure asks participants to remember an earlier list in the face of interference from a more recent list. Figure 1B illustrates the basic methodology. Participants study and recall a series of lists; after each list, they attempt to recall items from the *list-before-last* (Garlitch et al., 2023; Jang & Huber, 2008; Lehman & Malmberg, 2009; Sahakyan & Hendricks, 2012; Shiffrin, 1970; S. M. Smith, 1979; Unsworth et al., 2012a, 2013; Wahlheim & Huff, 2015; Wahlheim et al., 2016, 2019, 2017; Wahlheim & Garlitch, 2020; Ward & Tan, 2004).

In standard free recall, there is minimal RI, as recall follows immediately after the presentation of the study list. As such, it is not surprising that participants are generally better at recalling items from the most recent list than from the list before last. In everyday life, episodic retrieval rarely follows so closely on the heels of the initial experience. More likely, the experience and the retrieval suffer from interference related to the encoding and retrieval of other memories. Despite this increased interference, participants typically make an order of magnitude more correct responses from the target list than intrusion responses from the intervening list (Jang & Huber, 2008; Sahakyan & Hendricks, 2012).

Shiffrin (1970) introduced this procedure as an empirical challenge to classic theories which assumed RI reflected a combination of competition between new and old associations and unlearning of old associations caused by the learning of new associations (see Postman & Underwood, 1973, for a review). If studying intervening items causes unlearning of target items, then quadrupling the intervening list length should worsen target-list recall. Shiffrin (1970) found that the length of the intervening list did not affect target-list recall rates (Jang & Huber, 2008; Sahakyan & Hendricks, 2012; Unsworth et al., 2012a; Ward & Tan, 2004) and interpreted this invariance as strong evidence against unlearning (but see Smith, 1979). Rather, Shiffrin suggested that any difficulties participants experience retrieving LBL items arose from a failure to retrieve the context of the target list.

Rather than causing unlearning of old information, the prevailing evidence suggests that performing recall between the target and the intervening lists protects target-list memory from RI from the intervening list. For example, if a brief pause, rather than a recall period, separates study of the target and intervening lists, then target list recall decreases as the length of the intervening list increases (Jang & Huber, 2008; Ward & Tan, 2004). When varying the nature of the task participants performed between lists, the consistent level of target-list recall level across intervening list-length only emerged for between-list tasks requiring episodic memory (Jang & Huber, 2008, see also Divis & Benjamin, 2014). These results underscore the role of episodic memory retrieval in influencing RI, such that stronger RI effects reflect a loss in accessibility of relevant retrieval cues.

Theoretical accounts of data obtained using this procedure often assume that participants associate a list-specific context with the target list and that the nature of the intervening material (e.g., intervening list length, recall, or pause between lists) influences accessibility to the list context (Jang & Huber, 2008; Lehman & Malmberg, 2009). However, these models leave open the question of how the intervening material influences accessibility. As an attempt to explain these processes, Lohnas et al. (2015) developed a model which augments the retrieved-context theory of memory (Howard & Kahana, 2002; Polyn et al., 2009; Sederberg et al., 2008) with a post-retrieval editing process, similar to that proposed by Anderson and Bower (1972). This model, CMR2, assumes that each studied item is associated with a slowly drifting temporal context state. This context state serves as the recall cue, and retrieving an item leads to the retrieval of its associated temporal context from study. In standard free recall, CMR2 only overtly recalls an item retrieved from memory if the retrieved item’s associated temporal context is sufficiently similar to the current context state. However, in the LBL procedure, CMR2 filters recalls by requiring a retrieved item’s context to not be too similar to the contextual state prevailing at the start of recall, because then this item would most likely be from the intervening list. At the same time, an item’s associated context cannot be too dissimilar to the current context, because then the item is most likely from a more distant list.

CMR2 can account for several key findings from the LBL procedure. With a longer intervening list length, temporal context drifts further away from the target list context, and thus it is less likely that CMR2 can use its current context state to recall target list items. As a result, CMR2 predicts reduced target-list recall for longer intervening list lengths when there is only a pause between lists. However, this dynamic changes when there is a list-before-last recall period between each list. In this case, after the target list ($n-1$) is presented, but before the intervening list (*n*) is presented, CMR2 searches for items from an earlier list ($n-2$). This contextual retrieval clouds the current context state, generally making it more difficult for the model to retrieve target list items during the next recall period. Importantly, this increased difficulty is due to retrieval from the earlier list, rather than the presentation of the intervening list, which allows CMR2 to capture the finding that intervening list length doesn’t affect target list recall performance.

However, not all evidence favors CMR2’s approach. Sahakyan and Hendricks (2012) posited that, if more items are recalled in the period between the target and intervening list, then this should cloud the context representation further and thus reduce target-list recall. Sahakyan and Hendricks (2012) manipulated the delay between study and test of the list before the target list, and found that increasing delay length reduced the number of recalls from the list preceding the target list. Yet in the subsequent recall period, delay length did not influence target list recalls nor intervening list intrusions. Sahakyan and Hendricks (2012) took these results as evidence against the importance of context retrieval during each intervening recall test. In response, Lohnas et al. (2015) proposed that the search process itself might be sufficient to cloud the current context state, regardless of how many items participants overtly recall.

Some findings from the dual-list free-recall procedure have also been posed as a challenge to CMR2. In this procedure, participants study two lists in succession, and are then asked to recall either the first or the second list (Epstein, 1972; Unsworth et al., 2013; Wahlheim & Huff, 2015). When the first list is targeted, dual-list free recall becomes a list-before-last procedure. Using this procedure, Wahlheim et al. (2019) found that recall probability for the first list did not differ whether an intervening task involved short-term memory (a 2-back task) or long-term memory (exemplar generation). This was highlighted as a challenge to CMR2, in that retrieval from long-term memory might be expected to trigger more contextual retrieval than the cognitive operations involved in the short-term memory task. Future work is needed to resolve this challenge, e.g., by developing the model to account for short-term memory task performance, to determine whether the retrieval operations necessary to perform the task require less contextual updating than the exemplar generation task.

The Wahlheim et al. (2019) study also characterized individual differences in performance, finding that participants with higher working memory capacity exhibited a reduced primacy effect in list-before-last recall after performing the intervening long-term memory task than after the short-term memory task. Wahlheim et al. (2019) proposed that the results were consistent with the long-term memory task imposing greater context change for participants with higher working memory capacity. They proposed this as a second challenge to CMR2, in that CMR2 does not assume the existence of a separate short-term memory store. However, as they note, CMR2 has yet to be developed to account for individual differences, especially with respect to short-term memory, so further work is needed to resolve this debate.

Despite these challenges, CMR2 can overcome some shortcomings of prior models developed to explain list-before-last recall. Notably, the unique nature of the LBL procedure challenges theories that lack a mechanism of list differentiation. Lohnas et al. (2015) solved this problem by assuming that participants cue recall using the state of context at the end of the intervening list and then use context-similarity to filter out inappropriate recalls from the more recently studied list. This assumption also aligns well with the tendency to report intervening list intrusions earlier in the recall period (Lohnas et al., 2015; Wahlheim et al., 2017). Healey and Wahlheim (2023) proposed a more direct process of targeting the appropriate list, by assuming that participants encode then partially reinstate a start-of-list context (similar to Lewandowsky & Murdock, 1989). Direct reinstatement of the target-list context then promotes recall of other target-list items. Healey and Wahlheim (2023) found that implementing this post-encoding, pre-production reinstatement (PEPPR) mechanism in CMR2 yielded more accurate predictions of recall accuracy than the CMR2 model variant from Lohnas et al. (2015). Importantly, this suggests that participants can target items experienced at specific times using salient cues. In standard free recall, such cues may be less critical because the participant’s (or model’s) state just prior to recall initiation serves as a strong cue to current list items. By contrast, the LBL procedure highlights the additional challenges of recalling items beyond one’s most recent experiences.

Although Healey and Wahlheim (2023) assessed their PEPPR mechanism with a single set of list-lengths and a pause between lists, other models also assume automatic target-list context reinstatement, which varies with intervening list length and the task performed between lists (Jang & Huber, 2008; Lehman & Malmberg, 2009). As a more direct investigation of target-list context reinstatement, Unsworth and colleagues tested the account that the target-list context can be isolated and reinstated by examining recall performance and recall latencies. They found that patterns of performance were most consistent with an account assuming that the reinstated target list contains noise from intervening list and prior list items which have shared context features with target-list items (Unsworth et al., 2012a, 2013). Building on prior work examining recall probabilities, they found that recall latencies increased in the presence of an intervening list but did not change further when the intervening list length was varied. This provides further evidence that intervening list items compete with target items during recall, but how these items compete is independent of intervening list-length when there are recall periods between lists. Taken together, these theoretical accounts of the LBL task enrich our understanding of how the reinstatement of a target list context can be affected by and insulated from both proactive and retroactive interference from other experiences.

## The recall-by-category (RBC) procedure

In the recall-by-category (RBC) procedure, each list consists of words drawn from a small number of categories. In traditional free-recall procedures, the list itself serves as the retrieval cue, but here the category serves as a secondary cue, and participants must recall only those list items that belong to the cued category. In other words, the RBC procedure provides participants with a retrieval cue that directly specifies a subset of items from the target list. This can be contrasted with the inter-list repetition and the LBL procedures, which both tax participants’ abilities to focus retrieval on the target list. In the inter-list repetition procedure, the overlapping content of the lists makes it more difficult to selectively retrieve items from the most recent list. In the LBL procedure, the presentation of the intervening list and recall of earlier lists both interfere with recall of the target list.

The RBC procedure can be contrasted with the more standard procedure of categorized free recall (e.g., Pollio et al., 1969; Wingfield et al., 1998). In both categorized free recall and RBC, lists comprise *N* exemplars drawn from each of *M* categories for a total of $M \times N$ items (e.g., a 20-item list may consist of five animal names, five types of furniture, five occupations, and five gemstones). In categorized free recall, the participant is asked to recall all of the items without regard to the category structure of the list. In other words, categorized free recall manipulates the structure of the study list, but uses standard free-recall instructions; as such, we do not include categorized free recall as a specialized recall procedure. A number of procedural variants blend features of RBC and categorized free recall. For example, providing category names for reference during free recall (e.g., Gershberg & Shimamura, 1995; Mathews, 1954; Wingfield et al., 1998), or providing a subset of categorized study items for reference during free recall (Lewis, 1971; Slamecka, 1968; Wood, 1969). The theoretical contributions of the RBC procedure are best understood in terms of the many insights gained from categorized free recall, motivating a concise review.

With both procedures, the category identities of studied items have powerful effects on memory performance, consistent with the idea that category is a powerful retrieval cue (B. H. Cohen, 1966). Because all study items are targeted simultaneously in categorized free recall, this procedure allows examination of category clustering, whereby items from the same category tend to be recalled successively (Roenker et al., 1971). The inter-response times for these within-category recall transitions are generally faster than for between-category transitions (Patterson et al., 1971; Pollio et al., 1969; Wingfield et al., 1998). Category clustering suggests that when an item is retrieved, its category identity becomes part of the retrieval cue influencing the subsequent memory search, making other members of that category more accessible (Raaijmakers & Shiffrin, 1980; Sirotin et al., 2005). Generally speaking, more items are retrieved from lists with strong category structure than weak or no category structure (Cohen, 1963), and more items are retrieved when inter-item associative strength is greater (Bousfield & Cohen, 1955; Deese, 1959a), consistent with the idea that category information supports effective memory targeting. This effective targeting can be seen when category names are provided at test, in terms of improved recall performance and the elimination of retroactive interference from intervening study lists (Tulving & Psotka, 1971). The use of category information to guide recall may have a degree of strategic control (Becker & Lim, 2003), as the strength of category clustering can be manipulated with an instruction for participants to focus on pre-existing semantic relationships between the study items during memory search (Healey & Uitvlugt, 2019).

Categorized free recall tasks have also been used to demonstrate how the category structure of a study list interacts with the list’s temporal structure: Presenting items from a given category in blocks usually yields better performance than intermixed presentation (Cofer et al., 1966; Dallett, 1964). Shorter inter-item distances between category members generally improve performance (Glanzer, 1969; Puff, 1974) though larger inter-item distances between category members can make it more likely that at least one item from a category will be remembered (Borges & Mandler, 1972). Studying lists with all items drawn from a single category improves performance but disrupts temporal organization relative to lists with no category structure (Hong et al., 2024). In recent work, the memory disruptions associated with shifts between categories in lists with blocked presentation have been used to understand the memory disruptions seen more generally with other types of boundaries between events (DuBrow & Davachi, 2013; DuBrow et al., 2017; Zacks et al., 2007).

The RBC procedure deviates from the standard categorized free recall procedure by creating separate mini-retrieval phases, each cued by a specific category name. In perhaps the most influential study involving the RBC procedure, Tulving and Pearlstone (1966) found a substantial increase in the number of items recalled with RBC compared to free recall (see also Dong, 1972). They interpreted this to mean that many item memories are only temporarily inaccessible to the participant during free-recall search, becoming accessible given an appropriate retrieval cue, in this case, the category name. They also found that the number of items recalled with RBC was inversely related to the number of study items (the set size) associated with the category, suggesting competitive interference between the items targeted by a given category cue (Raaijmakers & Shiffrin, 1980). Evidence suggestive of between-category interference was seen in another study; more items were recalled from a targeted category when it was the only one targeted, as compared to a condition where two categories were targeted within the same recall period (Epstein, 1969, 1970). Proactive output interference from prior RBC recall attempts has been consistently observed, where the number of items recalled per category steadily decreases across a series of RBC periods (Dong, 1972; Nickerson, 1984; Roediger & Schmidt, 1980; A. D. Smith et al., 1970; A. D. Smith, 1971).

The preceding examples focus on tasks using taxonomic categories to cue memory search, but the RBC procedure can be used with any feature of the studied materials that defines a specific subset of the studied items. For example, Earhard (1967) used the word’s first letter to define the category set, varying the number of study items beginning with a given letter, replicating the set-size interference effect described above. Dalezman (1976) defined a set of three temporal categories: Participants first targeted a subset of serial positions (first five, last five, or middle five) before shifting to free recall of all items, demonstrating that items first targeted for recall enjoyed a performance advantage and interfered with items retrieved later. Roediger III and Tulving (1979) used an inverted form of RBC (in which participants were told which categories to exclude from search) to demonstrate that performance was not improved relative to a free-recall condition, suggesting that participants used post-retrieval monitoring (rather than memory targeting) to ensure correct responses during the inverted RBC period.

In a particularly clever variant of RBC, Watkins and Peynircioǧlu (1983) had participants study items drawn from three very distinct task contexts, such that neighboring study items were associated with different tasks. Three RBC periods followed the list, each targeting one of the three task contexts. Watkins and Peynircioǧlu (1983) found that in the later recall periods, even though participants had already engaged in recall of items from one of the other contexts (presumably a quite distracting mental operation), there was a robust and persistent recency effect. This result was surprising, as studies of the delayed free recall (DFR) paradigm (Glanzer & Cunitz, 1966; Postman & Phillips, 1965) suggested that the first recall period should have disrupted the recency effect for the later recall periods.

**Fig. 3:**
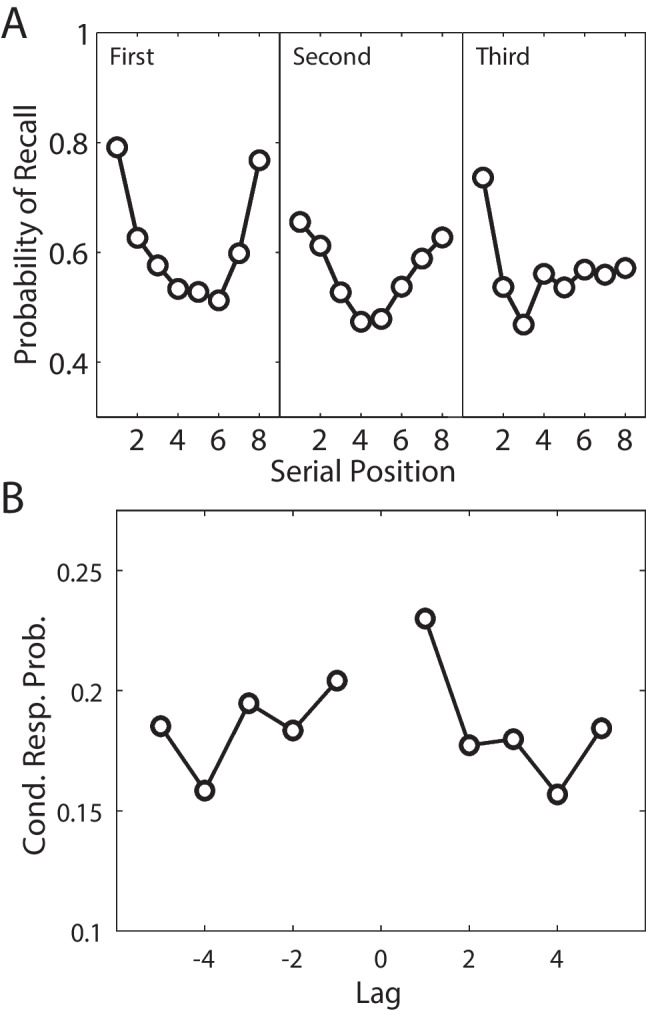
Recency, primacy and contiguity in the Recall-by-Category procedure. **A.** Within-category serial position curves for the three recall-by-category trials reported by Polyn et al. (2011). From left to right, each panel depicts the probability of recalling the eight studied items belonging to the cued category from the first, second, and third recall period, respectively. **B.** Lag-CRP analysis averaged across the three recall-by-category periods, showing a temporal contiguity effect for within-category recall transitions, despite those items being separated by multiple other-category items on the original study list. Adapted from Polyn et al. (2011)

Polyn et al. (2011) used the RBC procedure to examine how a strong semantic cue (a category label) interacts with the temporal structure of a study experience. Items from three categories were interspersed on study lists, such that same-category items never appeared in adjacent serial positions. Like Watkins and Peynircioǧlu (1983), they found a recency effect persisting across multiple retrieval periods (Fig. 3a). Furthermore, within each recall period, they found temporal contiguity effects spanning the non-adjacent study positions occupied by same-category items (Fig. 3b). This suggests that the strong semantic relations among the categorized words allowed the memory system to bridge the temporal gaps separating the categorized words from one another, resulting in both a persistent recency effect and a long-range contiguity effect. CMR proposes that semantic and temporal information are combined during memory search to support retrieval of specific memories (Hong et al., 2024; Morton & Polyn, 2016; Polyn et al., 2009). This combination should allow CMR to capture these RBC phenomena, though the model has never been used to directly simulate these data.

Categorized free recall and the RBC procedure have also proven useful in the domain of clinical neuropsychology. Incisa Della Rocchetta and Milner (1993) showed that patients with damage to their left frontal lobe were impaired (relative to matched comparison participants) on categorized free recall, but that this impairment was not apparent when memory was tested using the RBC procedure. They interpreted this as supporting the view that frontal cortex is important for the strategic use of memory (Moscovitch, 1992), as the provision of category cues in the RBC procedure does some of the strategic work that would otherwise be done by the frontal system (Jetter et al., 1986). Unsworth et al. (2012b) examined these ideas in healthy participants, showing that the provision of category labels (via RBC) reduces the difference in performance seen between groups with high and low estimates of working memory capacity. These performance differences were not seen for smaller category set sizes, suggesting that participants with lower working memory capacity are more negatively affected by within-category interference, or cue overload. Finally, participants with high working memory capacity performed worse in an RBC condition relative to free recall, likely because they forged temporal associations between study items that were useless when items were probed one category at a time.

The RBC procedure allows an experimenter to more directly influence the retrieval processes that focus search on particular memories. The category cue can be used to isolate sets of items from interference from the broader list (and from other lists), and to demonstrate the competitive interference between items from the same category. Raaijmakers and Shiffrin (1980) demonstrated in simulations of the Tulving and Pearlstone (1966) results that these key effects are captured by a simplified version of the Search of Associative Memory (SAM) model that removes the short-term buffer component of the model. In their categorized free recall simulations memory search is initiated with a list-wide contextual retrieval cue; when an item is retrieved, the retrieval cue incorporates the category identity of the just-recalled item. The model then engages in a search for members of that category until it reaches a threshold of failed recall attempts, where recall failures include retrieving an already recalled item and retrieving a member of a different category. After hitting that threshold, the model returns to its list-wide contextual retrieval cue, searching for a member of another category. In their RBC simulations, retrieval of each category identity is guaranteed, but otherwise, the recall process uses similar dynamics. The model captures both within- and between-category interference effects, and how these effects differ in the free recall and RBC periods.

The Raaijmakers and Shiffrin (1980) work laid a solid theoretical foundation for our understanding of the cognitive dynamics associated with the RBC task, but a number of important challenges remain, regarding how temporal structure and category structure of a study experience interact during memory search. Work with multinomial models has made progress on this front by estimating separate parameters associated with the storage versus retrieval of category clusters, as a function of the spacing of the items on the study list (Riefer & Batchelder, 1988). These models have been applied to a handful of behavioral phenomena (e.g., memorability of pairs of categorized items), rather than providing a comprehensive account of many recall-related phenomena. Sirotin et al. (2005) developed the eSAM model, which provided a comprehensive account of many temporal and semantic effects seen with standard free recall procedures, using vector space models of semantic relatedness to estimate the strength of inter-word semantic associations. However, eSAM underpredicted the degree of category clustering in simulations of a study by Kahana and Wingfield (2000), raising the possibility that an explicit representation of category structure (above the item-level interrelations captured by the vector space model) would help improve the model’s predictions.

The RBC procedure hints at rich interactions between temporal and semantic structure in memory search. Successive RBC periods exhibit a persistent recency effect that is seemingly less fragile than that observed in immediate and delayed free recall, and RBC recall sequences show temporal contiguity effects that span more than just immediately adjacent study items. These phenomena suggest a multidimensional retrieval cue that simultaneously targets items with both temporal and semantic information. The Context Maintenance and Retrieval (CMR) model describes a retrieval cue with both semantic and temporal components (Lohnas et al., 2015; Morton & Polyn, 2016; Polyn et al., 2009). Hong et al. (2024) used CMR to simulate several versions of categorized free recall tasks, showing how category structure can modulate temporal organization dramatically depending how the categorized items are spaced throughout the study list. CMR seems well suited to capture the persistent recency and contiguity effects seen in the RBC procedure, though this modeling work remains to be carried out. Such model developments may also incorporate how participants target their search based on category cues, as well as how they use post-retrieval monitoring if an item from an incorrect category is retrieved (see also “The Externalized Free Recall Procedure”).

## The final free recall procedure

Each procedure discussed above deviates from traditional free recall in manipulating how people search their memories for the target items. The inter-list repetition procedure creates overlapping information across distinct lists, making it harder to focus retrieval on the most recent list. The list-before-last procedure forces participants to target items from an earlier list but without any explicit categorical or semantic cue to aid their search. In the recall-by-category procedure, each list mixes items from distinct categories, but at recall, participants must focus their search on just those items that belong to the cued category. Whereas the inter-list repetition procedure requires participants to use contextual information to overcome semantic interference, the recall-by-category procedure requires participants to use semantic information to overcome contextual interference.

**Fig. 4:**
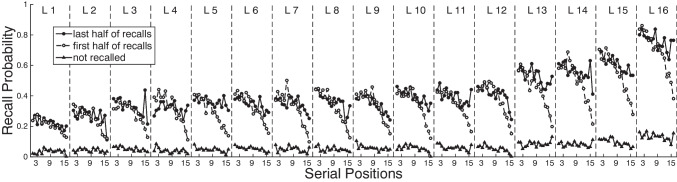
Final-free recall as a function of recency of encoding. Final Free Recall probability is shown for three classes of items: Those recalled in early output positions in immediate-free recall (*open circles*), those recalled in late output positions (*filled circles*), and items that were not recalled initially but were recalled during final-free recall (*filled triangles*). We observe a positive long-term recency effect across lists, and a negative-recency effect within lists. The negative-recency effect appears greatest for the items initially recalled in early output positions. Adapted from Kuhn, Lohnas and Kahana (2018)

This section considers the final free recall (FFR) procedure, which expands the timescale of episodic targeting considered with the other procedures. Here, after participants study and recall many distinct lists over the course of a multi-list session, the experimenter asks them to freely recall as many items as they can remember from across all of the previously studied lists. In FFR, participants freely search their memories without regard to list boundaries, and they remember a surprisingly large number of items, further demonstrating excellent retention of information from list to list, even when the final test is given as a surprise (R. L. Cohen, 1970; Craik, 1970; Craik et al., 1970; Dalezman, 1976; Darley & Murdock, 1971; Howard et al., 2008; Kuhn et al., 2018; McCabe & Madigan, 1971; Unsworth, 2008). An important feature of this procedure is that the final test is not a pure measure of memory for the initial study event; rather, it is a measure of memory for both the initial study event and the initial test event, which may or may not have resulted in recall of the target item.

Figure 4 illustrates data from an FFR experiment in which participants first studied and freely recalled 16 lists (each comprising 16 common words) and then attempted final free recall of all 256 words (Kuhn et al., 2018). Figure 4 shows the probability of final recall as a function of recency of encoding, considering both the list that each item came from and the item’s serial position within that list, for a total of 256 ($16 \times 16$) possible positions. Because retrieval of an item can produce substantial learning, and because spacing between items affects long-term retention, Kuhn et al. (2018) partitioned the recall data into three classes of items: Those initially recalled in early output positions (first half of recalls), those initially recalled in late output position (second half of recalls), and those not initially recalled.

Figure 4 illustrates three major effects: First, participants recalled more items from recent than from remote lists. Second, participants rarely recalled items that they failed to initially recall. Third, participants exhibit a pronounced negative recency effect within each list, whereby items from end-of-list positions tend to be poorly recalled during the FFR test. This negative recency effect is enhanced when items from those end-of-list positions are initially recalled in early output positions, and is attenuated when those items are initially recalled in later output positions.

In a classic FFR experiment, Craik (1970) reported a negative recency effect similar to the one illustrated in Fig. 4. He considered two possible explanations: 1) Participants failed to recall end-of-list items because those items received fewer rehearsals while in short-term memory and thus they did not form strong long-term memory representations of those items needed to support final free recall performance. 2) Participants failed to recall end-of-list items because those items would tend to have shorter study-test lags during initial recall and thus would not benefit from the well-known spacing effect wherein participants exhibit superior retention of repeated items when they are widely spaced (e.g., Melton, 1970; Siegel & Kahana, 2014). A series of papers provided support for Craik’s first explanation (Craik et al., 1970; Dalezman, 1976; McCabe & Madigan, 1971), leading researchers to disregard and ultimately forget Craik’s second explanation. However, Kuhn et al. (2018) presented several data points supporting the spacing account over the rehearsal account of negative recency. They showed that the probability of final free recall increased monotonically with the spacing between the study of an item and its initial recall. Because participants tend to recall end-of-list items early in output, these items will benefit less from spacing than earlier list items, and those end-of-list items recalled early will have the shortest spacings of all, and thus the lowest recall levels, as seen in Fig. 4. Kuhn et al. further examined final recall following lists in which participants performed a continual distractor free recall task. According to the rehearsal-based account, negative recency arises because end-of-list items receive fewer rehearsals than mid-list items, and as such, the end-of-list items have weaker associative representations in long-term memory. As CDFR should disrupt rehearsal, they predicted substantially reduced negative recency in this condition. Contrary to the rehearsal account and in line with the spacing account, Kuhn et al. found strong negative recency in the CDFR condition.

In FFR transitions may be between items of the same list or between items in different lists, and in the former case it is not surprising that FFR transitions between words studied in the same list show a clear contiguity effect (Howard et al., 2008; Loaiza & McCabe, 2012; Unsworth, 2008). Because FFR tests the same items as in the initial recall test, it is also perhaps not surprising that these transitions share similarities to the subjective organization of participants’ initial recalls (Lohnas, 2024). At the same time, participants exhibit greater temporal clustering in FFR than in the initial free recall test (Lohnas, 2024; Sadeh et al., 2018). This is consistent with increased subjective organization across successive tests of the same list (Tulving, 1962), but is all the more noteworthy because in FFR participants have the potential to transition between items studied in different lists.

In standard free recall procedures, temporal organizational analysis characterizes the web of associations linking items within a study list. Analysis of across-list transitions in FFR gives insight into how these within-list temporal structures relate to broader temporal structures spanning the entire experimental session.

One may ask whether contiguity effects extend across list boundaries when participants make transitions among items studied on separate lists. In particular, if participants store unique list contexts for each list, any two lists may be equally similar to each other. Furthermore, participants may cluster their recalls by list without benefiting from the shared temporal information among neighboring lists. By contrast, if participants associate temporal contexts with each list, as CMR2 assumes, then participants should be more likely to transition between items studied in neighboring lists.

**Fig. 5:**
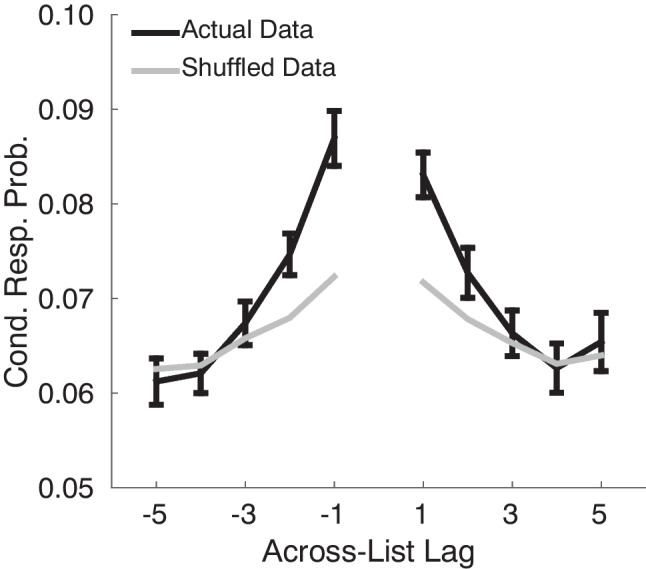
Across-list transitions during final free recall show a contiguity effect. The *black curve* shows the conditional response probabilities computed from the actual data. The *gray curve* shows the conditional probability from surrogate data produced by randomly shuffling the order of recalls. Adapted from Healey, Long, and Kahana (2019)

Figure 5 illustrates the probability that participants transition, in final recall, between items studied on different lists as a function of the *lag* between the lists (a lag of $+1$ indicates a transition between an item studied on List *i* and an item studied on List $i+1$). This *across-list contiguity analysis* considers only transitions between correct recalls from one list to correct recalls of a different list, and it controls for the fact that it becomes less likely to transition to a particular list as the number of items already correctly recalled from that list increases. Following Howard et al. (2008), a surrogate (shuffled) data set was constructed to control for the effects of recency and autocorrelated goodness of encoding (each of which could generate a greater tendency for neighboring items to be recalled successively). These surrogate data (gray line in Fig. 5) exhibit a substantially smaller contiguity effect than seen in the non-shuffled data.

We have seen how studies of final free recall have provided useful data for understanding the effects of rehearsal, spacing, and contiguity on subsequent recall. At a broader level, final free recall provides valuable information on long-term retention of memoranda in tasks requiring only short-term retention, as often the participant is unaware that the session will end with an FFR test. By asking participants to freely recall a large pool of items, one can observe retrieval dynamics under conditions where participants recall dozens of items. In a multi-session experiment in which participants, at the start of each session, freely recalled all words learned across all prior sessions, Katerman et al. (2022) observed average recall rates exceeding 100 words on the final experimental session. This allows for a rich examination of how the dynamics of free recall characterized with single lists extend to longer time scales.

Data from the FFR procedure also requires preservation of information across multiple interfering lists. For items recalled in both the initial and final free recall test, the consistency in recall transitions suggests retrieval of memories of initial recall sequences, not just the sequences of studied items (Lohnas, 2024; Sadeh et al., 2018). Thus, FFR results place constraints on output encoding in models like CMR2, even though presently CMR2 assumes that the successive recall of two items serves to strengthen their associations in the current context. In addition, the preservation of across-list information poses a challenge to theories which only assume direct item-to-item associations, as they would have more difficulty explaining the recency and contiguity exhibited at the list level (Howard et al., 2008; Lohnas, 2024; Unsworth, 2008). By contrast, CMR2 predicts both within-list and across-list transitions because items studied (and recalled) closer in time share more similar contexts. However, thus far such CMR2 predictions have only been explored in prior-list intrusions with standard free recall (Lohnas et al., 2015), and CMR2 may face challenges when assuming a single temporal context representation underlies both within- and across-list effects. Nonetheless, a retrieved context model assuming representations of context on multiple timescales can account for final free recall effects (Howard et al., 2015). This underscores the broader advantage of the FFR procedure to examine representations of memories beyond a single list. Combined with the implications for recency items and output encoding, the FFR procedure has advanced understanding of episodic recall on multiple timescales.

## The overt-rehearsal procedure

In this and the next section, we consider procedural variants of free recall aimed at unmasking the covert rehearsal processes hypothesized to occur during learning and the covert retrievals we may filter out during recall. In the overt-rehearsal procedure, Rundus and Atkinson (1970) asked participants to say, out loud, everything that came into their minds as they tried to memorize the words on a study list for a free recall test. Rundus and Atkinson then counted the number of overt rehearsals each item in the list received. They found that early list items received the greatest number of rehearsals and that participants often continued to rehearse these items until the end of the list. Further, the number of rehearsals an item received predicted its eventual recall. This finding, coupled with participants’ preferential rehearsal of early list items, supported theories attributing the primacy effect to participants’ rehearsal strategies (Atkinson & Shiffrin, 1968; Raaijmakers & Shiffrin, 1980).

Using the overt-rehearsal procedure, Brodie and Murdock (1977) examined the relation between presentation rate and the primacy effect. Previous work has shown that slower presentation rates produce stronger primacy effects. Brodie and Murdock showed that with a slow (5 s per item) presentation rate, early list items also tend to be rehearsed later in the study list than with a fast (1.25 s per item) presentation rate. They argued that by rehearsing early list items later in the list, those items benefit from greater *recency*. Thus, the rehearsal process not only leads participants to devote more time to some items than to others, it also makes items from earlier list positions appear later in the sequence of rehearsals and thus makes them functionally more recent at the time of test.

Brodie and Murdock proposed that rather than examining recall as a function of the position of an item in the list, one should examine recall as a function of the position in which an item was last rehearsed. They thus contrasted nominal and functional serial positions. *Nominal serial position* refers to the position of the item in the list, as presented by the experimenter to the participant. *Functional serial position* refers to the position of the item in the participant’s sequence of rehearsals. Because a given item may be rehearsed many times during the study period, Brodie and Murdock decided that the most relevant position is that of the last rehearsal. Whereas Brodie and Murdock found pronounced primacy in the nominal serial-position curve, especially with a slow presentation rate, primacy vanished in their analysis of functional serial-position curves. Rather, the functional serial position curve showed a nearly continuous recency effect extending from the end of the list to the beginning. This analysis suggests that the primacy effect largely results from early list items being rehearsed more recently than middle items. Subsequent work by Ward and colleagues (e.g., Tan & Ward, 2000; Ward, 2002) replicated and extended these findings by showing that in a broad range of experimental conditions, the recency with which an item has been last rehearsed is the critical variable predicting its recall.

Murdock and Metcalfe (1978) examined the distribution of rehearsals following the presentation of each study item. They found that participants tend to rehearse recent items and items from the start of the list more than other items. The rehearsal mechanism thus generates a primacy and recency effect resembling those seen in free recall itself. This and other recall-like rehearsal phenomena led Laming to argue that rehearsals are essentially mini-recalls that participants make as they study the list (Laming, 2006, 2008). Laming’s view turns the analysis of rehearsals on its head. Rather than using rehearsal to explain recall, Laming argues that we should use our understanding of recall to explain rehearsal. In free recall, participants do not merely attend to the presented stimuli; rather, they use the study period to think about (i.e., recall) previously studied items. Data generated by the overt rehearsal procedure thus cannot establish a causal linkage between rehearsal and recall phenomena.

Though treated as separate stages of a memory task, encoding and retrieval occur in tandem. Elaboration during encoding entails retrieving associated representations from memory, and participants often deliberately rehearse earlier list items as an encoding strategy. The overt rehearsal procedure makes such retrievals explicit by asking participants to articulate every item that comes to mind during study.

Theories of memory either treat overt rehearsal as a separate encoding strategy (e.g., the rules governing the rehearsal buffer in the SAM model; Raaijmakers & Shiffrin, 1980) or as mini-recall periods between study events, as in Laming’s theoretical analysis (Laming, 2008, 2010). These opposing views reflect the criticism that using the overt rehearsal procedure to explain recall data with rehearsal data suffers from the chicken-and-egg problem. Unless we know why participants rehearse items the way they do, we can’t use their rehearsal to explain their recall. As a more systematic approach to examine the role of rehearsal, some studies include instructions for participants to rehearse in a particular manner (Murdock & Metcalfe, 1978; Tan & Ward, 2000), but exerting control in this way may substantially alter the nature of the rehearsals themselves (see Ward, 2024, for a discussion of this point). Even though the overt rehearsals produced by participants likely differ from natural retrieval processes during free encoding, overt rehearsals exhibit all of the usual properties of standard free recall, such as recency, primacy, and contiguity (Bhatarah et al., 2006; Friendly, 1979).

Whether overt rehearsal alters covert rehearsal processes or not, the procedure reveals patterns of behavior consistent with standard free recall and with participants’ use of covertly retrieved items to inform subsequent recall performance. More broadly, this relates to theoretical developments that thinking of items between experiences gives rise to new learning. Cohen and Kahana (2022) used the free recall process in CMR to model the way participants ruminate (covertly) about their previous experiences, thus drawing an analogy between free recall and covert retrievals. Similarly, Zhou et al. (2023) used CMR to simulate neural reactivation studied in animals during sleep and quiescent wake periods following learning. They also conceptualized covert retrievals as a free-recall process. The common factor, however, among all of these theories is that thinking of items between experiences gives rise to new learning. Wachter and Kahana (2024) developed a retrieved-context theory of financial decision-making in which covert thoughts become memories. The overt-rehearsal procedure offers an experimental methodology for uncovering these covert thoughts and showing how they interact with prior learning to shape future recall and behavior.

## The externalized free recall procedure

Ever since Melton and Irwin (1940), students of memory have recognized that failures of memory search appear not only as omitted responses but also as intrusions of items from outside of the target list. Deese (1959b) and Roediger and McDermott (1995) demonstrated how semantic associations cause participants to commit intrusions in free recall, often with high confidence. Zaromb et al. (2006) found a similar effect for temporally defined associations (see section “The inter-list repetition procedure”). However, under most circumstances, intrusions occur infrequently as participants likely censor themselves, omitting responses they deem inappropriate. Thus, the small number of *overt* intrusions that people commit during recall (typically around 5% of the total number of recalls) likely underestimates the role of intrusions in the recall process.

To study the influence of covert intrusions on recall, Kahana et al. (2005) introduced an externalized free recall (EFR) procedure adapted from an earlier method developed by Roediger and Payne (1985) and Bousfield and Rosner (1970). In the EFR procedure, after participants have become familiar with standard free-recall instructions, the experimenter asks them to say out loud all words that come to mind at the time of test, even if they think those words did not occur in the most recent target list. To separately examine the internal censoring process during recall, researchers using the EFR procedure may also ask participants to indicate when they have recalled an item that they believe was not on the most recent list by pressing a key immediately following recall of that item, indicating ”rejection” of that response. In the Roediger and Payne (1985) method, participants were asked to recall every word that came to mind, but they were not instructed to indicate which responses were errors. Most of the studies reviewed here aim to approximate standard free recall, tasking participants to recall as many words as possible from the most recently studied list and to indicate whether each recalled item occurred on the most recent list.

Some theories assume that participants generate more items than they actually recall, but apply an internal filter to only recall items from the current list. If this assumption is valid, the EFR procedure asks participants to recall *all* generated items while making their filtering criteria explicit (by rejecting responses deemed inappropriate to the target context). Lohnas et al. (2015) used EFR to evaluate the role of generate-recognize processes in their retrieved-context model of memory search (CMR2). Because CMR2 uses a context-comparison criterion to determine whether to recall a retrieved item, it is straightforward to simulate EFR data by allowing the model to recall any item it retrieves and then having the model reject any item that fails the context-comparison criterion. CMR2 assumes that both accepted and rejected retrievals update context.

**Fig. 6:**
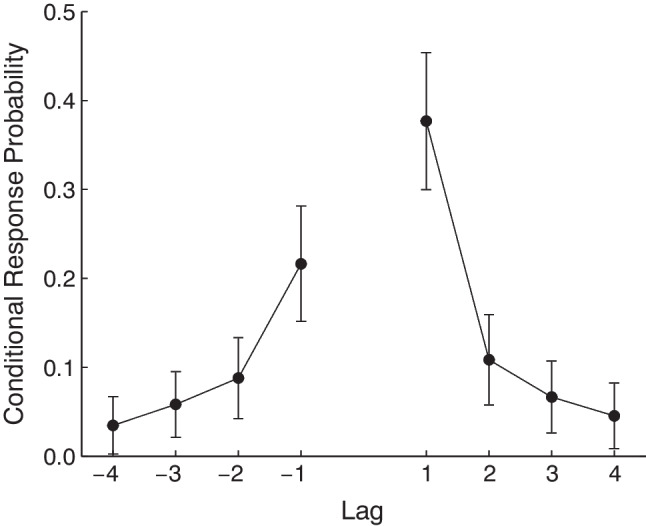
Temporal contiguity and prior-list intrusions (PLIs). Successive PLIs that came from the same original list tend also to come from neighboring serial positions on that list. Based on studies in the free-recall database (Sederberg et al., 2010) and a previous analysis reported in Zaromb et al. (2006). Adapted from Kahana (2012).

Comparing EFR and standard FR in a large dataset, Lohnas et al. (2015) demonstrated that EFR does not fundamentally change the processes that govern free recall, such as context updating. Although participants made many more intrusions in EFR, recall of correct items did not differ reliably between groups, suggesting that the increased recall of intrusions in EFR did not hinder the recall of correct items. In addition, the recency advantage in recall initiation and recall probability was preserved between the two tasks. Lastly, properties of recalled intrusions were preserved as well: Participants recalled as many prior-list intrusions (PLIs) in immediate free recall as the number of nonrejected PLIs in EFR. Lohnas et al. (2015) also showed that the same parameterization of the CMR2 model matched data from both EFR and standard free recall. These results buttress prior studies demonstrating that EFR instructions do not significantly interfere with the normal recall process (Kahana et al., 2005; Unsworth & Brewer, 2010; Zaromb et al., 2006).

CMR2 rejects retrieved items when the context they retrieve does not adequately resemble the target context, which, in the case of immediate recall, is the context at the time of test. Consequently, the model predicts a higher rejection probability for PLIs than for correct items and a higher rejection probability of PLIs from more distant than recent lists (Unsworth et al., 2010). Lohnas et al.’s EFR study verified these predictions, supporting the idea that participants use internal context states to filter out retrieved items.

To the extent that EFR provides a window into participants’ unfiltered thoughts during free recall, it enables a richer analysis of the recall dynamics leading to errors and demonstrates how erroneous recalls cue other memories. Consider data from an experiment where items repeat across lists (e.g., the inter-list repetition procedure). Correct recall of a repeated item should elicit retrieval of items from both of its associated lists, even though the items from earlier lists are errors. Zaromb et al. (2006) examined inter-list repetitions with an EFR procedure and found that when two PLIs occur in succession, and when they both came from the same list, they tend to come from neighboring list positions (Fig. 6). Similarly, in lists without repetitions, Unsworth et al. (2010) found that a PLI was less likely to be marked as an intrusion when it followed immediately after a correct response, as compared to PLIs that followed immediately after another PLI. They interpreted this finding as evidence that PLIs with a stronger connection to the target list context are more likely to be misinterpreted as correct responses. Unsworth et al. (2010) also found that rejection probabilities of intrusions were lower for early than late output positions, again suggestive of the stronger associations with the correct-list context used to initiate recall.

There is also much overlap in theoretical questions and experimental design between the EFR procedure versus the free recall procedure in which participants make a ”Remember” or ”Know” designation for each recalled item (Arnold & Lindsay, 2002; Garlitch et al., 2023; Guynn & McDaniel, 1999; Hamilton & Rajaram, 2003; Mickes et al., 2013; Read, 1996; Sadeh et al., 2015; Tulving, 1985). As in EFR, participants must make post-recall monitoring decisions regarding the likelihood that the just-recalled item was studied in the present list. This procedure borrows the designation from recognition tasks to distinguish between items remembered with specific details such as when the item was presented in the list, versus a general familiarity or knowledge that the item was studied without detailed information (Yonelinas, 2002). Tulving (1985) introduced this procedure to compare to rates of Remember judgments in recall tasks in which participants were provided with a unique category cue for each word, or a category cue as well as the word’s first letter. Participants exhibited the highest rates of remember judgments in the free recall task, and Tulving interpreted these results with the intuition that free recall places stronger demands on vivid episodic detail for correct recall. Subsequent studies provide evidence that recalled items classified as Remember exhibit properties consistent with greater levels of context retrieval (e.g., Hamilton & Rajaram, 2003; McCabe et al., 2011; Mickes et al., 2013), including greater temporal clustering (Sadeh et al., 2015) and correct recalls conditional on Remember judgments in earlier output positions (Garlitch et al., 2023). Procedures combining Remember/Know judgments with FFR also provide evidence that Remember judgments reflect greater context retrieval. Specifically, during the final test, participants are more likely to make Remember judgments for items recalled during the initial test (see also McDermott, 2006 Sadeh et al., 2018). This is consistent with the finding noted in *The Final Free Recall Procedure* that these items also boast greater temporal contiguity (Lohnas, 2024; Sadeh et al., 2018).

The EFR procedure has also clarified mechanisms underlying group differences in overall recall performance. In standard free recall, healthy younger adults with low working memory capacity recall fewer correct items and make more intrusions compared to younger adults with higher working memory capacity (Unsworth, 2007; Unsworth et al., 2010). The EFR procedure revealed that the increase in intrusions reflects reduced rejection probability and greater overt generation of PLIs. Similarly, older adults also recall fewer correct items and commit more intrusions than young adults in standard free recall (Kahana et al., 2002; Rhodes et al., 2019). As with low-capacity young adults, EFR reveals that older adults both generate more intrusions and exhibit lower rejection probabilities than younger adults (Kahana et al., 2005; Wahlheim et al., 2017). Further, in a procedure that had participants perform the EFR procedure and then make a confidence judgment on each recalled item, younger adults exhibited a greater difference in confidence judgments for correct recalls versus intrusions than older adults (Zaromb et al., 2006). Similarly, when making Remember/Know judgments for each recall, older adults provide more Remember responses to intra-list intrusions and fewer Remember responses to correct recalls than younger adults (Garlitch et al., 2023).

The EFR and Remember/Know procedures, like the LBL procedure, are both posited to require participants to evaluate each retrieval with respect to its correctness for the instructed list. From the perspective of CMR2, both tasks suggest that participants compare the context of each retrieved item to the context of the desired list. Further underscoring the similarities between these procedures, Wahlheim and colleagues examined performance in a series of studies which combined externalized recall procedures with recall of either the most recent list (List 2) or the list before last (List 1; Garlitch et al., 2023; Healey and Wahlheim, 2023; Wahlheim et al., 2017; Wahlheim et al., 2019). In the EFR condition, participants acknowledged more and rejected fewer across-list intrusions for List 1 than List 2 (Wahlheim et al., 2019). In a Remember/Know condition, participants made more Remember Judgments on across-list intrusions (Garlitch et al., 2023). These findings are consistent with the presence of List 2 context during List 1 recall, and highlight the increased challenges for the retrieval of more distant information. Generally, these findings also underscore that post-retrieval monitoring is a useful but imperfect mechanism to omit reporting of intrusions. Rejection rates in a recall task combining EFR and LBL procedures suggest that participants can also use list cues to initiate recall with the proper list, rather than just relying on post-retrieval monitoring (Healey & Wahlheim, 2023).

Nonetheless, the EFR procedure demonstrates that while performing recall, participants can exert post-retrieval monitoring to judge whether an internally retrieved item should be reported. The consistency between standard free recall and EFR suggests that participants may perform post-retrieval operations even during standard free recall (Lohnas et al., 2015). As such, successful recall of an item reflects both the item’s adequate encoding as well as the item’s ability to meet post-retrieval monitoring criteria (Koriat & Goldsmith, 1996). With respect to post-retrieval monitoring criteria, we reviewed several points consistent with the view that contextual similarity informs this process. Most notably, items with more similarity in temporal context to the current list were less likely to be rejected (Kahana et al., 2005; Lohnas et al., 2015; Unsworth et al., 2010; Wahlheim et al., 2019; Zaromb et al., 2006).

However, the imperfect relationship between context similarity and rejection rates may reflect other contributing factors to successful recall, whether based on search strategies prior to retrieval (see *The Recall-by-Category Procedure*) or monitoring strategies after retrieval (e.g., Jacoby & Hollingshead, 1990; Tulving & Thompson, 1973). CMR2 simulations of the EFR procedure can assess more directly inconsistencies with generate-recognize assumptions. To date, CMR2 assumes that each retrieved item evokes a consistent amount of context retrieval and consistent post-retrieval monitoring (Healey & Wahlheim, 2023; Lohnas et al., 2015), but future work may need to explore flexibility in these processes.

## Spatial-episodic memory procedures

Episodic memories unfold in both time and space (Kahana & Miller, 2013; Tulving, 2002), yet free recall procedures rarely manipulate the spatial context of memoranda. Several investigators have developed methods to study the joint contributions of temporal and spatial information to memory search by presenting words or objects presented in 2D arrays (e.g., Cortis et al., 2015; Gibson et al., 2011; Mandler et al., 1977; Naveh-Benjamin, 1987; Rothkopf, 1971). However, desktop VR paradigms have allowed researchers to study spatial contributions to episodic memory by having people experience information as they navigate a virtual environment from a first-person perspective (Herweg & Kahana, 2018).

**Fig. 7:**
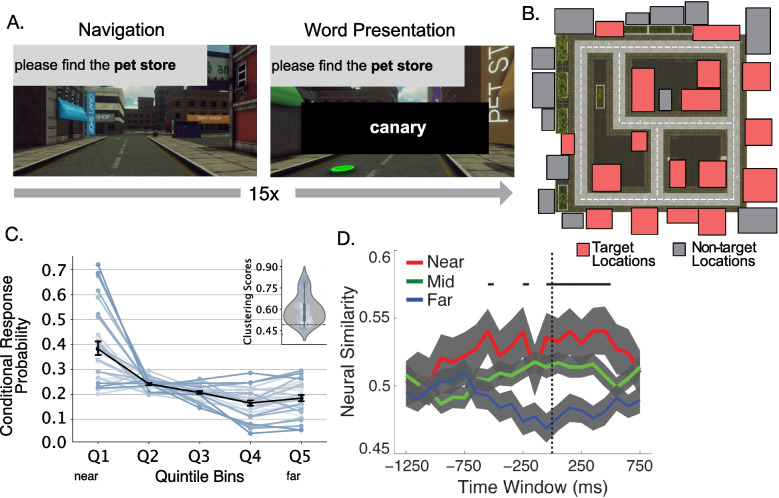
The Courier Task. **A.** Participants navigate along a courier’s delivery route in a virtual town, delivering an object to each business they visit (e.g., delivering a canary to the pet store). Participants subsequently recall each of the delivered objects, either freely or in response to cues. **B.** Overhead view of the town. **C.** Spatial clustering appears as an increased tendency to make transitions during recall to objects delivered in nearby spatial locations. Adapted from Dougherty et al. (2024). **D.** Miller et al. (2013b) showing the average neural similarity for near, middle, and far spatial distance bins between ensemble place-responsive cell activity during navigation and place-responsive cell activity during item recall as a function of time relative to recall onset, computed in overlapping 500-ms time windows (*x*-axis values indicate the center of the time window). *Shaded regions* indicate *SEM* across recalled items. A *horizontal bar* indicates statistically significant time points. Reprinted from Miller et al. (2013b), Copyright American Association for the Advancement of Science

This section discusses one such paradigm based on a navigational game introduced by Miller and colleagues (Miller et al., 2013a). In their *Courier* procedure, participants play the role of a courier in a 3D-rendered virtual town (Fig. 7). In each of a series of “delivery days,” participants navigate to a series of stores in the virtual town. Upon arrival at each store, the game informs participants of the identity of a list-unique object they delivered. After visiting the final store on the delivery route, participants attempt to freely recall the delivered objects from that delivery day. To decouple the effects of spatial and temporal proximity during encoding, the sequence of store deliveries on a given trial is generated without regard to their spatial proximity.

The interpolated store navigation between item deliveries, which typically takes 15–60 s, makes the task resemble continual-distractor free recall, where participants perform an interpolated distraction task between item presentations. Unlike continual-distractor free recall, where the distraction task bears no relation to the learning task, spatial navigation creates a context for the learned objects. Further, the spatial learning that occurs during navigation supports performance in the task by promoting accurate wayfinding and creating distinct spatial contexts for memoranda. Like continual-distractor free recall, the navigational demands of the Courier procedure may inhibit rehearsal strategies that participants might otherwise use to associate neighboring items. Yet, despite the navigational demands of the spatial-episodic procedure, participants achieve substantially higher recall rates than they do in continual-distractor free recall.

The spatial-episodic memory paradigm illustrates one use of desktop virtual reality (VR) in memory research. Desktop VR allows researchers to probe memory in a quasi-naturalistic setting while still providing precise experimental control. Whereas daily life generally does not involve learning a random list of words presented in rapid succession, the Courier procedure embeds these items in a spatial layout that does not change over trials, thus creating a contextual scaffold for the otherwise unrelated memories. Importantly, by manipulating spatial contextual information, researchers can directly study the role of context in organizing episodic memory. To this end, Miller et al. (2013b) asked whether participant recall sequences would exhibit both temporal and spatial clustering. They found that following the recall of a given object, the next recalled object was more likely to be from spatially proximate location. Much as conditional-response probability curves decrease as the temporal lag between memoranda increases, researchers using this task (Dougherty et al., 2024) have shown that the tendency to cluster items decreases as the Euclidean distance between those items increases (Fig. 7). These findings of spatial clustering in free recall, based on Euclidean distance, provide a link to cognitive map theories of memory (Herweg & Kahana, 2018; O’Keefe & Nadel, 1978).

To evaluate the cognitive map theory in the setting of the Courier task, Miller et al. (2013b) examined performance in neurosurgical patients fitted with hippocampal microwire electrodes. They sought to evaluate whether neural codes representing a spatial map form part of the spatiotemporal context hypothesized to underlie human episodic memory. By measuring neural spiking activity during gameplay, they found that a significant fraction of neurons responded selectively when participants navigated through particular regions of the virtual town, analogous to hippocampal *place cells* found in both rodents (O’Keefe & Dostrovsky, 1971) and humans (Ekstrom et al., 2003; Jacobs et al., 2010). During the spontaneous recall of an item, these place-responsive neurons exhibited firing patterns similar to the patterns they exhibited during navigation through the region of the town where the item was delivered (see Fig. 7). Miller et al.’s finding that spontaneous recall of an item reactivates its spatial context provides neural evidence for theories of episodic memory that postulate context reinstatement as the basis for recollection (Kahana, 2020). This finding further suggests that the spatial coding identified with the hippocampal place cell system is part of a more general engine of episodic memory in which items become associated with their spatiotemporal contexts and retrieval of items reinstates those contexts to help cue other context-appropriate memories.

Although students of memory typically assume that each item in a word list has a unique context, the contextual diversity within word list experiments is impoverished and contrived. The semantic structure of a word list determines the dimensions along which context varies in such experiments. For experiments that randomly choose words from a large lexicon, the semantic context for each word jumps around haphazardly through the list. For experiments that draw items from one or more semantic categories, the semantic context is relatively constant or jumps suddenly after a change in category. In contrast, the virtual navigation tasks, such as Courier, create a continuously varying spatial context for each encoded item. These tasks enhance the diversity of temporal context by introducing a secondary task (navigation) between each delivered item. Because efficient navigation between stores requires mental effort, this secondary task should reduce the degree of inter-item rehearsal, much like a continual-distractor free recall procedure (Bjork & Whitten, 1974; Howard & Kahana, 1999).

Despite the many differences between the Courier task and standard word list free recall, the Courier task exhibits the usual behavioral effects that characterize word list recall, including primacy, recency, temporal contiguity, and semantic similarity (Dougherty et al., 2024; Miller et al., 2013b). In addition, Courier gives rise to reliable levels of spatial clustering, even when participants have no specific reason to recall the spatial context of the studied items. Finally, Courier generally gives rise to fairly high levels of recall, possibly due to the rich contextual information surrounding each item.

Although we focused on one particular procedure in this section, we see the technology of virtual reality as opening up the door to a wide range of naturalistic recall paradigms. Of course, virtual reality environments have been used for decades to study spatial navigation abilities and memory for the spatial layout of a particular environment (Bird & Burgess, 2008; Burgess et al., 2002; Montello et al., 2004). But virtual reality has also been used for decades to examine how spatial context affects memory for other aspects of events encountered within that spatial context. This includes examinations of how person, time, and object memories are affected by spatial context (Burgess et al., 2001; King et al., 2002, 2004; Spiers et al., 2001), how free recall of one’s thoughts during a prior virtual navigation task illuminates brain activity patterns observed during navigation (Spiers & Maguire, 2006), and how neuronal firing patterns indicate subsequent memorability of object-location associations (Miller et al., 2018; Tsitsiklis et al., 2020). We are excited to see how future use of virtual reality methods will help bridge the gap between controlled laboratory methods and the study of memory in naturalistic settings.

## Summary and synthesis

Each of the seven specialized recall procedures reviewed here expands the boundaries of traditional recall paradigms in various ways. For example, while standard free recall uses a unique set of items on each trial, the inter-list repetition procedure challenges memory by including, within each list, a subset of items learned on prior lists. By eroding the contextual boundaries between lists, this procedure challenges participants to focus their retrieval on the target list while inhibiting neighbors of the repeated items experienced on earlier lists.

The combination of these specialized recall procedures can yield additional theoretical benefits. For example, the externalized free recall procedure provides evidence that recall of an inter-list repetition’s second presentation can evoke temporal context from its first presentation (Zaromb et al., 2006). According to CMR2, the repeated item’s retrieved temporal context incorporates the items neighboring its first presentation. This assumption has yielded verified predictions of free recall dynamics in a single list with spaced repetitions. After recalling a repeated item, participants’ transition patterns suggest that the second presentation of the repeated item triggered contextual retrieval of the first presentation (Lohnas, 2025b; Siegel & Kahana, 2014). This allows the model to predict remote transitions between the neighbors of the repeated item’s first presentation and its second presentation (Siegel & Kahana, 2014).

The list-before-last procedure challenges memory in a different way. Whereas standard free recall asks participants to retrieve the set of most recently experienced items, the list-before-last procedure asks participants to avoid recalling those prepotent items and instead focus their memory search on those items experienced on the preceding list. Because these lists typically draw items from a common pool of words, temporal context is well-posed to serve as a cue that can be used to disambiguate the lists in memory. How this temporal context may interact with a unique list-level cue remains an open question, and underscores the importance of taxing memory retrieval by targeting more than just one’s most recent experiences.

The recall-by-category procedure changes the scope of targeting from an entire list to a subset of items that share semantic features. This matches many situations in daily life where you recall, e.g., all of the vegetables you bought on a recent shopping trip, or all of the interactions you had with a particular coworker. Recall by category allows researchers to manipulate the targeting process that occurs during memory search. Doing so has led to insights into the nature of within-list interference, and has provided evidence for the persistence of recency and contiguity across multiple recall periods.

Rather than narrowing the set of contextually appropriate responses, as in the recall-by-category procedure, the final-free-recall procedure broadens the set to include all lists experienced by the participant. Here, participants attempt to recall items irrespective of list membership as long as they occurred during the more general context of the experiment. During final free recall, participants typically recall a much larger number of items but a smaller fraction of the items initially recalled on any given list. If one conducts final free recall across multiple days of an experiment that uses a single large word pool, participants can freely recall more than 100 unique words in a single recall period (Katerman et al., 2022). Final free recall reveals how recency influences recall at short and long time scales and how temporal contiguity spans many items and multiple lists (Figure 5; Howard et al., 2008; Lohnas, 2024; Sadeh et al., 2018).

The overt-rehearsal procedure sheds light on the strategic processes occurring during intentional memory encoding. Participants frequently report verbally rehearsing words, and such rehearsals would be recorded in memory and shape subsequent recall. The overt-rehearsal procedure specifically asks participants to verbalize these rehearsals and shows that the frequency and temporal pattern of vocalized rehearsals predict important features of recall. Data produced in overt-rehearsal experiments have helped memory scientists address various theoretical questions about memory, as summarized in a review by Ward (2022).

The externalized free recall procedure also changes the contextual targeting of memories. But rather than manipulating the structure of the lists, this procedure asks participants to recall any item that comes to mind rather than to inhibit contextually inappropriate responses. The procedure further asks participants to report when they recognize a retrieved item as coming from the wrong context. Externalized recall thus reveals information about memory search that would be obscured when participants know that they must avoid recalling items that do not belong to the target list.

In the spatial-episodic memory procedure, participants experience items within a spatial context. In this free-recall variant, participants play a game in which they learn the layout of a computer-generated virtual town as they navigate it from a first-person perspective, playing the role of a courier delivering objects to businesses. This game exploits modern computing technology to break out of the traditional discrete item presentation format that has characterized memory research since the creation of the memory drum at the turn of the 20th century. Using a quasi-naturalistic format of item presentation, the Courier game creates both a spatial context for each memorandum and a natural separation between memoranda governed not by the timing of the experiment but by the participant’s navigational behavior. The dynamics of recall in this procedure reveal the joint influence of temporal and spatial context on memory retrieval. The quasi-naturalistic nature of encoding, in which a complex navigational task separates the encoding of items, reveals the degree to which principles of memory search generalize beyond the setting of traditional word list experiments.

Free recall models like CMR have not been extended to capture the influence of spatial context on memory search. There are several successful models of spatial memory that characterize the construction of internal representations of both allocentric (environment-centered) and egocentric (person-centered) representations of spatial context (Byrne et al., 2007; Madl et al., 2015). The CMR framework was developed to account for the simultaneous deployment of multiple context representations containing different kinds of information, including task, emotionality, and reward value (R. T. Cohen & Kahana, 2022; Horwath et al., 2023; Polyn et al., 2009; Talmi et al., 2019). A profitable avenue of future simulation work could use a spatial model to define the state of spatial context for CMR, allowing for a hybrid model that captures spatial and episodic behaviors in a unified framework. Such an effort would find a stable foundation to build upon, with modeling work demonstrating that the principles of retrieved-context models can account for the development and firing properties of hippocampal place cells, which represent specific locations in an environment (Howard et al., 2005).

More generally, we have used retrieved context models such as CMR and CMR2 as a framework linking the standard free recall procedure to the specialized recall procedures in this review. Although CMR2 has not yet been developed to account for the inter-list repetition or spatial-courier procedures, results from these procedures are generally consistent with participants’ use of temporal context cues to elicit correct recalls in standard free recall. These same temporal context cues elicit errors in the inter-list repetition procedure, as the temporal context of earlier presentations evokes retrieval of items from prior lists. CMR2 simulations of the LBL procedure also suggests that context cues promote recall of items from the incorrect (intervening) list (Healey & Wahlheim, 2023; Lohnas et al., 2015). However, as noted above, work remains to capture contributions of short-term and working memory, as well as individual differences in these abilities.

CMR2 remains to be extended to several of the recall procedures here, but is well posed to do so. CMR2’s assumptions regarding semantic structure could enable the model to capture benchmark effects from the RBC procedure. At the same time, the model’s proposed interactions between temporal and semantic context should enable it to account for long-range contiguity and recency effects (Hong et al., 2024; Polyn et al., 2011). Such temporal context representations could also support CMR2’s ability to capture the broader set of cues supporting across-list effects in FFR. As a first step in this direction, CMR2 accounts for the temporal contiguity between two successively recalled PLIs in free recall (Lohnas et al., 2015), yet more work needs to be done to capture the dynamics of FFR where such recalls are correct rather than intrusion errors.

Yet CMR2 simulations of the EFR procedure – consistent with empirical findings – suggest that intrusion errors share several notable properties with correct items, most notably that they support context retrieval (Lohnas et al., 2015; Unsworth et al., 2010; Zaromb et al., 2006). However, work remains to develop CMR2’s pure context retrieval account to incorporate other established metacognitive processes (e.g., Healey & Wahlheim, 2023; Jacoby & Hollingshead, 1990; Kelley & Sahakyan, 2003; Tulving & Thompson, 1973). Just as intrusion errors raise the question of how similar their dynamics are to those of correct recalls, so too can we raise the question regarding similarities between overt (and covert) rehearsals and to what extent they serve as endogenous recalls during study. CMR2 can be developed to simulate each of these possibilities, providing further insights into interactions between encoding and retrieval. As described above, several developments to the CMR framework have already advanced our understanding of how covert thoughts during encoding affect subsequent memory.

Standard free recall, serial recall, and paired associate procedures (we only focused on free recall) have generated valuable data for over a century and will continue to have currency in our quest to understand memory at a psychological, biological, and even societal level (Kahana, 2012). The standard versions of these procedures arose from a particular set of historical pressures. Starting around 1960, short-term memory procedures, such as immediate free and serial recall, rapidly generated data showing how variables such as list position, modality, presentation rate, repetition, and spacing influence recall performance. Theories emerged to explain existing data and generate predictions to be verified in further experimental studies (Murdock, 1974). Laboratory computers that replaced slide projectors and memory drums (in the 1970s) provided even greater experimental control. It is not an accident that these classic procedures survived and thrived. At the same time, researchers have long recognized limitations of these standard procedures which can be overcome by methodological innovations. The present review considered several procedural variants that aim to overcome specific limitations of traditional free recall, as described above.

We can better understand the similarities and differences of different recall tasks by using consistent procedures and materials across tasks, and examining common patterns in empirical results. Recently, there has been increasing interest in constraining experimental design to be as similar as possible across participants in free recall and serial recall (e.g., Grenfell-Essam & Ward, 2012; Grenfell-Essam et al., 2017; Solway et al., 2012; Ward et al., 2010). These studies yield remarkably similar patterns of recall by initiation, serial position and output position, and similar effects of manipulating experimental variables such as list-length, modality, and test expectancy. Furthermore, constraining computational models to be as similar as possible across free and serial recall allows us to leverage these empirical commonalities and develop unified theories of performance across these tasks (Brown et al., 2007; Farrell, 2012; Gunn & Polyn, in press; Lohnas, 2025a). Relevant to CMR2 development, retrieved context models can account for findings from serial recall, whole report, and copy typing tasks (Logan, 2021; Logan & Cox, 2023; Lohnas, 2025a; Osth & Hurlstone, 2023). Future advancements may also benefit from incorporating features of the specialized procedures described here. More work remains, whether in free recall specifically or recall tasks more broadly, to characterize recall in episodic memory tasks.

We see particular value in studying people’s ability to target memories outside of the context of a recently presented list and in the presence of overlapping cues. Memory is not a tabula rasa and theories need to tackle the challenging question of how participants focus their search on the most recent list while filtering out memories from prior lists (Lohnas et al., 2015). Whereas the list-before-last procedure requires focusing on items specific to a list or temporal context, the recall-by-category procedure demands focus on a subset of list items. Both tasks challenge models of free recall like CMR2, which generally initiate recall using the cues available at the start of the recall period. In the recall-by-category procedure, although these cues are no longer as effective, the exogenous cue of the relevant category can still facilitate correct recall. In contrast, the list-before-last procedure imposes demands on effective endogenous cues. Future work remains to assess how these cues are encoded and retrieved, including in CMR2. Healey and Wahlheim (2023) highlights the utility of using a formalized model like CMR2 to distinguish between different possible assumptions of how participants encode and utilize their cues.

We see the analysis of recall errors as providing important insights into the process of contextual targeting as seen in intrusions coming from prior lists (Zaromb et al., 2006) and intervening lists (Jang & Huber, 2008; Wahlheim et al., 2019). To assess intrusions, it is helpful to encourage participants to report any item that comes to mind and mark those items they deem as errors, as in the externalized recall procedure. Although this procedure provides support for the CMR2 proposal that participants rely on similarity in temporal context to omit intrusions that come to mind during recall, it also reveals contributions from other processes and representations. Incorporating these other factors would benefit CMR2 and retrieved-context theory. Finally, we see modern computing technology as offering us the ability to advance our understanding of memory by embedding the demands of a traditional memory experiment within a more naturalistic situation, thus helping to ensure that the memory mechanisms uncovered in the laboratory generalize to our understanding of people’s everyday lives (Dougherty et al., 2024).

### Conclusion

The open-ended instructions of standard free recall provide unique insights into episodic memory organization. We show how seven specialized procedures with relatively simple yet profound changes to standard free recall provide further insights. When compared to standard free recall, these procedures all dramatically alter what the participant must retrieve or report. These procedures also all advance and challenge theories developed from standard free recall. Indeed, we draw similar conclusions from several procedures about critical future directions for current theories. Thus, these specialized free recall procedures highlight both the strengths of free recall as well as the importance of considering complementary procedures to comprehensively characterize episodic memory.

## Open Practices Statement

Not applicable, as this theoretical review does not have any associated data or materials to be made available.

## Data Availability

Not applicable
